# Improving patient understanding of intravenous zoledronic acid treatment using an audiovisual resource

**DOI:** 10.1136/bmjoq-2026-004451

**Published:** 2026-11-10

**Authors:** Abigail Phillips, Lewis Hardy, Maximillan Paley, Lauren Gurr, Clare Hunt, Pamela Leventis

**Affiliations:** 1 Care of The Elderly Department, Maidstone and Tunbridge Wells NHS Trust, Tunbridge Wells, UK; 2 Kent and Medway Medical School, Canterbury, UK; 3 Rheumatology Department, Maidstone and Tunbridge Wells NHS Trust, Tunbridge Wells, UK

**Keywords:** Informed Consent, Shared decision making, Patient-centred care, Patient education

## Abstract

**Background:**

Zoledronic acid is a first-line treatment for osteoporosis following hip fracture, reducing further fracture risk and associated morbidity and mortality. However, counselling practices can vary between clinicians, and older adults with visual impairment may have difficulty accessing written resources. This service evaluation assessed whether a novel audiovisual resource could improve patient’s understanding of the benefits and risks of zoledronic acid compared with standard written information.

**Methods:**

This evaluation was conducted at a UK district general hospital between October 2024 and March 2025. Patients aged over 60 years admitted with low-impact femoral fractures who were recommended intravenous zoledronic acid were included. All patients received standard verbal counselling, the Royal Osteoporosis Society factsheet and a novel narrated audiovisual resource. Participants rated how much each resource helped their understanding of the benefits and risks of zoledronic acid using 5-point Likert scales (1=‘did not help at all’, 5=‘helped a lot’). Statistical analysis was performed using Wilcoxon signed-rank for paired comparisons and Mann-Whitney U tests for subgroup analysis, with rank-biserial correlation (r) for effect size.

**Results:**

36 patients were analysed (mean age 80.9 years, 62% female). 25% (n=9) had recorded visual impairment. Wilcoxon signed-rank testing showed significantly higher understanding score for both treatment benefits (Z=−3.86, p<0.05, r=0.84) and risks (Z=−3.43, p<0.05, r=0.83) after viewing the audiovisual resource compared with the written factsheet. Subgroup analysis demonstrated a slightly greater effect size in visually impaired participants (r=0.90) compared to those without visual impairment (r=0.80–0.83). Mann-Whitney U tests comparing the two-subgroup revealed no statistically significant differences in patient reported understanding (p>0.05), indicating that the audiovisual resource was equally beneficial across both groups.

**Conclusion:**

The audiovisual resource significantly enhanced patient understanding of zoledronic acid, particularly among visually impaired participants. Incorporating multimedia resources into standard counselling may improve accessibility, equity, and patient engagement in orthogeriatric care. Further evaluation should examine long-term knowledge retention, adherence, and wider clinical implementation.

## Introduction

 One of the foundations of good medical practice is informed consent, ensuring patients are involved in and educated on their treatment options.^1^ Verbal guidance supplemented by information leaflets is the standard of practice in drug counselling, but written information may be less accessible to visually impaired patients.^2^

Neck of femur fractures are a common sequela of falls in older adults and are associated with significant morbidity and mortality.^3^ Intravenous zoledronic acid is often recommended as a bone protection agent.^3 4^ However, zoledronic acid is associated with several risks and therefore patients must be properly counselled before its prescription.^5^ Patients presenting with neck of femur fractures are typically older and often have cognitive and visual impairments,^6^ thus ensuring informed consent can be challenging.

We aimed to compare a novel, clinician-developed audiovisual resource to the standard written information leaflet in aiding patients’ understanding of the benefits and risks of intravenous zoledronic acid as secondary fracture prevention treatment.

## Methodology

This service evaluation was conducted by the orthogeriatric team at a UK district general hospital from October 2024 to March 2025, with the aim of comparing patients’ understanding of the risks and benefits of intravenous zoledronic acid from a novel audiovisual resource and a standard written information leaflet.

Patients 60 years and older who had been admitted with a low-impact femoral fracture, and who had zoledronic acid recommended within their bone protection plan were included. Patients with moderate to severe cognitive impairment who lacked capacity to consent to participation were excluded. If participants had a form of visual impairment, this was recorded for subgroup analysis.

Capacity to consent to participation was assessed clinically. Patients considered to have capacity were given an explanation of the purpose of the evaluation and what participation would involve and were then asked whether they wished to participate. All participants received verbal counselling from a clinician before reviewing the patient information resources.

Participants received a written information leaflet created by the Royal Osteoporosis Society and access to a novel audiovisual narrated PowerPoint created by our team. Participants were asked which resource they wished to review first. After viewing both resources, participants were asked to complete a questionnaire consisting of 5-point Likert-style questions assessing their self-reported understanding of both the benefits and risks of treatment.

A Shapiro-Wilk test demonstrated that the data were not normally distributed; therefore, the Wilcoxon signed-rank test was used for paired comparisons between resources. Matched-pairs rank-biserial correlation was used to estimate effect size, with 95% CIs calculated using percentile paired bootstrap resampling (50 000 resamples). Mann-Whitney U tests, with adjustment for tied ranks, were used for comparison between visually impaired and non-visually impaired participants.

## Results

Of the 75 patients identified for this study, 36 were included. Participant age ranged from 63 to 97 years and 62% were female. Of these participants, 25% (n=9) had a form of visual impairment, the most common being cataracts and presbyopia.

Self-reported understanding was higher after the audiovisual resource than after the written information for both treatment benefits and risks in the whole cohort. For benefits, the mean score increased from 3.61±1.29 to 4.67±0.79 (Wilcoxon W=4.5; p=0.000089; rank-biserial r=0.961; 95% CI 0.842 to 1.000). For risks, scores increased from 3.58±1.29 to 4.53±0.88 (Wilcoxon W=4.0; p=0.000524; rank-biserial r=0.948; 95% CI 0.789 to 1.000) (table 1).

**Table 1:** Mean Likert score±SD and paired comparisons after reviewing the two resources in the whole cohort and visually impaired and non-visually impaired subgroups

Group	Outcome	Written information (mean±SD)	Audiovisual resource (mean±SD)	Wilcoxon p	Rank-biserial r
Whole cohort (n=36)	Benefits	3.61±1.29	4.67±0.79	0.000089	0.961
	Risks	3.58±1.29	4.53±0.88	0.000524	0.948
Visually impaired (n=9)	Benefits	3.11±1.76	4.78±0.44	0.0269	1.000
	Risks	3.22±1.79	4.89±0.33	0.0394	1.000
Non-visually impaired (n=27)	Benefits	3.78±1.09	4.63±0.88	0.00101	0.942
	Risks	3.70±1.10	4.41±0.97	0.00462	0.910

The 95% CIs for the primary whole-cohort rank biserial correlations were 0.842–1.000 for benefits and 0.789–1.000 for risks.

Subgroup analysis therefore showed the same direction of effect in both visually impaired and non-visually impaired participants, with large effect sizes. There was no statistically significant difference in self-reported understanding between the two groups. For the written resource, Mann-Whitney U=98, p=0.385 for benefits and U=109, p=0.650 for risks. For the audiovisual resource, U=119.5, p=0.940 for benefits and U=88, p=0.137 for risks.

## Discussion

The results of this evaluation showed a statistically significant increase in self-reported understanding with the audiovisual resource, with large effect sizes, supporting its use as an adjunct to clinician-led counselling. Notably, the benefit was observed for all participants, not just those with visual impairment. An audiovisual resource is also easy to incorporate into routine clinical practice, making it a low-cost and practical intervention.

A meta-analysis of 29 randomised controlled trials by Farrell *et al* found that audiovisual information aids improved immediate information recall compared with standard consent processes (Standardised Mean Difference (SMD) 0.64, 95% CI 0.45 to 0.85), although longer-term findings were heterogeneous.^7^ Similarly, an updated systematic review by Glaser *et al* found statistically significant improvements in comprehension in 15 of 27 audiovisual interventions, while highlighting substantial heterogeneity between interventions and outcome measures.^8^ Our findings therefore align with the broader literature while extending this observation to an older orthogeriatric population.

There are several limitations to this service evaluation, the most significant being the small sample size due to the high exclusion rate caused by a high prevalence of cognitive impairment in this cohort. Understanding was assessed using self-reported Likert scores rather than objective testing of knowledge or recall. Consequently, the study cannot establish whether the audiovisual resource improved the validity or overall quality of informed consent itself. Longer-term retention was also not assessed. Resource presentation order was also not formally randomised creating a potential carry-over or ordering effect. In addition, verbal counselling was delivered using a standard documentation prompt rather than a verbatim script, therefore variation between clinicians may have influenced participants’ subsequent ratings.

Despite these limitations, the findings suggest that audiovisual patient information resources may promote inclusive, accessible consent discussions in orthogeriatric care. Audiovisual resources should be regarded as an adjunct to, rather than a replacement for, individual clinician-led discussion and shared decision-making. Larger studies could be conducted to support these findings and explore effects on objective knowledge, longer-term retention, treatment uptake and adherence.

**Learning points**

- Audiovisual patient information resources may improve self-reported understanding of treatment risks and benefits in orthogeriatric patients.
- Audiovisual resources may provide an accessible adjunct to clinician-led consent discussions but should not be considered a substitute for the consent process itself.
